# From My Note Book, No. 1

**Published:** 1849-01

**Authors:** B. T. Whitney


### FROM MY NOTE BOOK.—No. I.

BY DR. B. T. WHITNEY.

We often observe some peculiar freak of nature in the development of various organs of the human system; and however interesting any of them may be to the inquirer after science or facts, cause and effect, none can engage the attention of the Dental Surgeon with more interest than those pertaining to the teeth.

For our gratification, as well as the permanent good of our patients, when called upon to remedy a defect in the development of nature, it becomes necessary to look into the cause as well as the malformation. This is often very obscure, and can only be fully understood by careful investigation with extensive observation.

After understanding the cause of such a freak, we may not always be able to induce nature to the perfect completion of her work, yet, in adapting artificial appliances, we may be much guided in the final and perfect accomplishment of the matter, and avoid the chagrin and mortification that ignorance must beget. Many of the most peculiar character, we may seldom see, even in an extensive practice; and are prepared to meet them only from our knowledge of recorded cases. These we cannot expect to find in the text books on general practice.

I do not presume that finite power could have remedied the cases I here record; but note them on account of their interest to me, and hope it may call out those who may have others of greater interest, or an explanation of the cause, from some one who may have given this subject greater attention.

In 1843, a gentleman, aged 24, of Athens, Pa., called on me for a partial upper set of artificial teeth. I removed several decayed teeth and roots, and in due time adapted a plate with artificial substitutes. He continued to wear the plate for about a year with much satisfaction, when he began to complain of the plate not fitting the gums as well as formerly; but thought he had bent it in cleaning. On examining the mouth and plate,

I found that a great change had taken place in the alveolar ridge, unaccountable to me, as the process was entirely absorbed before adapting the plate. I succeeded in bending the plate, however, so as to be worn for a while, when he called again, complaining of the same difficulty.

I now found a protuberance directly in front, over the incisive fossa, which was growing rapidly. Some weeks after, a tooth appeared through the gums, when I cut a notch in the plate, removing the two front incisors for its reception. It grew very slowly, and for a year he wore the imperfect artificial set, when I adapted a new plate—he refusing to have the strange intruder removed, but nursed it with care and affection, anxiously looking for "a few more of the same sort." Though a very troublesome guest, yet I would like to have possessed myself of it, and would gladly have given it a place with other *distinguished teeth.*

The general appearance of the tooth was that of a full-sized cuspidatus, firm and healthy. When I prepared the mouth, the left one I found much decayed, and removed. The right one he said he had had extracted long before, as well as one of the incisors. The others were very irregular and much decayed. I could learn nothing definitely of the gentleman in regard to the early condition of the teeth, only that they were very irregular and imperfect, and decayed early, and that he had had several extracted. He had lost the right first bicuspidatus, and my opinion was, that he had called that the *eye-tooth;* and that this untimely stranger was really the *eye-tooth* of the second set, and had been forced from its natural position by its neighbors, or had been refractory, and in the labyrinth had lost its own proper place and become entangled between the fangs of the other teeth, and finally confined there until these extractions gave it an opportunity to make its exit.

In 1840, I inserted the upper cuspidati for the lady of the Rev. Mr. R., aged about 30, now of Rochester, N. Y. The teeth that had just been lost, I learned, were very small, and unlike the others in general appearance. For many years they had been loose; and that on their removal, the roots were much

*decayed;* but could not learn whether she had shed these teeth or not. I pronounced them the deciduous teeth; and the prominence in the jaw above went to confirm me in the belief that the permanent cuspidati were still imbedded there. But why had they not appeared?

The protuberance became more and more prominent for a year; and I confidently expected the appearance of the prominent teeth. In consequence of the change in the form of the mouth, it became necessary to alter the plate; which she still continues to wear without farther trouble.

In 1840, I inserted the two under front incisors for Mr. N. P., of Norwich, N. Y., aged 25. The temporary set were entire; but these two had never been replaced. The space was still as great as the two teeth would have occupied; the lateral incisors not having approximated each other, as would be expected in such a case. Otherwise, the set was entire, and very regular, though much decayed. The gum at this point had the appearance of having had the teeth extracted, and the alveolar process entirely absorbed.

Up to the fall of 1847, when I saw him last, no change had taken place in the jaw: the plate was still good.

More anon.

CLARKSVILLE, Tenn., Dec. 9, 1848.

